# Prediction of the efficacy of group cognitive behavioral therapy using heart rate variability based smart wearable devices: a randomized controlled study

**DOI:** 10.1186/s12888-024-05638-x

**Published:** 2024-03-06

**Authors:** Zexin Lin, Junjie Zheng, Yang Wang, Zhao Su, Rongxin Zhu, Rongxun Liu, Yange Wei, Xizhe Zhang, Fei Wang

**Affiliations:** 1 Early Intervention Unit, Department of Psychiatry, The Affiliated Brain Hospital of Nanjing Medical University, Nanjing, P.R. China; 2 Functional Brain Imaging Institute of Nanjing Medical University, Nanjing, P.R. China; 3 School of Biomedical Engineering and Informatics, Nanjing Medical University, Nanjing, Jiangsu China; 4 Henan Key Laboratory of Immunology and Targeted Drugs, School of Laboratory Medicine, Xinxiang Medical University, Xinxiang, P.R. China; 5 Department of Psychiatry, The Second Affiliated Hospital of Xinxiang Medical University, Henan Mental Hospital, Xinxiang, Henan China

**Keywords:** Group cognitive behavioral therapy, Prediction, Heart rate variability, Depression, Anxiety

## Abstract

**Background:**

Depression and anxiety are common and disabling mental health problems in children and young adults. Group cognitive behavioral therapy (GCBT) is considered that an efficient and effective treatment for these significant public health concerns, but not all participants respond equally well. The aim of this study was to examine the predictive ability of heart rate variability (HRV), based on sensor data from consumer-grade wearable devices to detect GCBT effectiveness in early intervention.

**Methods:**

In a study of 33 college students with depression and anxiety, participants were randomly assigned to either GCBT group or a wait-list control (WLC) group. They wore smart wearable devices to measure their physiological activities and signals in daily life. The HRV parameters were calculated and compared between the groups. The study also assessed correlations between participants’ symptoms, HRV, and GCBT outcomes.

**Results:**

The study showed that participants in GCBT had significant improvement in depression and anxiety symptoms after four weeks. Higher HRV was associated with greater improvement in depressive and anxious symptoms following GCBT. Additionally, HRV played a noteworthy role in determining how effective GCBT was in improve anxiety(*P* = 0.002) and depression(*P* = 0.020), and its predictive power remained significant even when considering other factors.

**Conclusion:**

HRV may be a useful predictor of GCBT treatment efficacy. Identifying predictors of treatment response can help personalize treatment and improve outcomes for individuals with depression and anxiety.

**Trial registration:**

The trial has been retrospectively registered on [22/06/2023] with the registration number [NCT05913349] in the ClinicalTrials.gov.

**Condensed abstract:**

Variations in heart rate variability (HRV) have been associated with depression and anxiety, but the relationship of baseline HRV to treatment outcome in depression and anxiety is unclear. This study predicted GCBT effectiveness using HRV measured by wearable devices. 33 students with depression and anxiety participated in a trial comparing GCBT and wait-list control. HRV parameters from wearables correlated with symptoms (PHQ, PSS) and GCBT effectiveness. Baseline HRV levels are strongly associated with GCBT treatment outcomes. HRV may serve as a useful predictor of efficacy of GCBT treatment,facilitating personalized treatment approaches for individuals with depression and anxiety.

**Supplementary Information:**

The online version contains supplementary material available at 10.1186/s12888-024-05638-x.

## Introduction

Depression and anxiety are prevalent and debilitating conditions that significantly affect children and young adults [1–4]. Previous surveys have consistently highlighted the significant impact of depression and anxiety on adolescents and young adults, with prevalence rates ranging from 23 to 39% [5]. The college years represent a critical period characterized by increased vulnerability to a wide range of mental health challenges. Recent evidence indicates rising rates of depression, anxiety, and suicidality among college students [6, 7], highlighting mental health as an escalating and serious public health concern on college campuses [8, 9]. While common psychiatric conditions often emerge during late adolescence and early adulthood [10], a growing number of college students with subthreshold mental health issues face challenges in accessing effective help due to the insufficient number or duration of symptoms to meet DSM-IV or ICD-10 criteria [11]. Consequently, these unresolved issues can potentially develop into major depressive disorder (MDD) and generalized anxiety disorder (ANX) [12, 13], leading to an increased risk of suicide and functional impairment [14, 15]. The high rate of comorbidity between anxiety and depression among college students, ranging from 4.60 to 12.98% [16], can increase the risk of developing MDD and ANX, and is associated with more severe symptoms, impaired work performance, and poorer treatment outcomes [4, 17]. Therefore, early intervention is crucial to prevent the progression of these disorders and to mitigate their associated negative consequences, there is an urgent need to regulate key factors and develop more effective prevention and intervention strategies to address these growing mental health concerns among college students.

Common preventative intervention methods for depression and anxiety include medication, psychotherapy and physiotherapy. Cognitive behavioral therapy (CBT) has received widespread attention due to its highly structured nature and good therapeutic effect [18]. And CBT practiced in a group format offers a cost-effective option that can be delivered to many participants with a minimum of time and staff numbers. Group cognitive behavioral therapy (GCBT) can achieve its therapeutic effect by enhancing emotion regulation and cognitive control to promote self-regulation, which can help individuals better manage emotional fluctuations in daily life, resulting in further improvement of depressive and anxious symptoms [19]. However, despite various forms of CBT have shown effectiveness in adults, whether this kind of early intervention has positive effects on the adolescent and young adults remains unclear [20].

In previous studies on the efficacy of GCBT, the assessment of GCBT effectiveness primarily relied on therapists and psychiatrists, who employed patient questionnaire surveys and clinical observations for identification and monitoring. Additionally, the lengthy duration of CBT may exacerbate the lag effect in retrospective evaluations, further compromising their authenticity. Relying solely on subjective description, without objective biomarkers included in the assessment process, hinders an accurate reflection of the clinical severity of the evaluation and the participants’ response to efficacy. The applications of biological markers can provide more comprehensive and rigorous analysis, enabling the identification of participants who are more likely to benefit from treatment. One validated measure of autonomic function is heart rate variability (HRV), which refers to the variation in the intervals between heartbeats and is considered an objective biological marker with the potential to predict efficacy [21]. Previous studies have shown that HRV can be used as a biomarker to assess the response of antidepressants to anxiety and depression. Patients with higher high frequency of heart rate variability spectrum (HF-HRV) tend to have better prognoses than those with lower HF-HRV in anxious depression [22]. Changes in low frequency (LF) to high frequency ratio (LF/HF ratio) in response to emotional stimuli were positively correlated with a reduction in depression symptoms during fluoxetine treatment [23]. Additionally, Shapiro et al. showed that MDD responders had significantly increased HF-HRV and decreased LF-HRV during yoga therapy, compared to non-responders [24], which shows that HRV is closely related with clinical efficacy of different intervention. HRV is closely related to dysregulation in certain neural circuits of mental illness, especially amygdala and the medial prefrontal cortex (mPFC). Based on the neural circuit mechanisms and imaging of cognitive-behavioral therapy (CBT), it has been revealed that CBT effectively modulates abnormal neural circuits in patients by specifically targeting and modulating the amygdala and anterior cingulate cortical (ACC) circuits [25].Therefore, we put forward a hypothesis that HRV, a potential biological indicator, could predict the effectiveness of GCBT.

In the randomized control study, we investigated the predictive utility of pre-treatment HRV in effectiveness of GCBT in reducing depression and anxiety symptom among college students. The study seeks to examine the relationship between HRV and improvement clinical symptoms. By comparing traditional survey measures with HRV data, our study determine the additional value of HRV recorded in the real-world using wearable devices in GCBT. This approach helps identify individuals more likely to benefit from GCBT, facilitating a more efficient utilization of medical resources. The findings have implications for improving treatment approaches for subthreshold mental health and advancing personalized interventions.

## Method

### Participants

This randomized controlled study was conducted at Xinxiang Medical University in China, focusing on the recruitment of college students. Utilizing the WeChat subscription platform, participants were engaged in completing mental health screening questionnaires and providing demographic information, including age, gender, lifestyle, and health characteristics [26]. Validated self-report questionnaires, such as the PHQ-9, GAD-7, and PSS-14, were employed to assess depression, anxiety, and stress. Different cutoff points and approaches were utilized in pre- and post-treatment assessments to define participants with depression and anxiety, and the changes in these results will be considered as the primary outcomes for evaluating the efficacy of GCBT. The inclusion criteria required participants to have PHQ-9 or GAD-7 scores above 5, be aged between 16 and 25 years old, agree to undergo four-week treatment, and have no medication or psychotherapy in the three months prior to enrollment. Exclusion criteria encompassed no history of DSM-IV diagnosis of schizophrenia or other mental disorders, no evidence of head injury or organic brain disease, no diagnosis of substance dependence, and no high suicide risk or heart disease. Written informed consent was obtained from all participants. A total of 58 participants completed all online questionnaires and wore activity trackers from 7:00 to 21:00 for a minimum of 28 days during the treatment period. Eligible and consenting participants were randomly assigned to either the GCBT group or wait-list control (WLC) group using a web-based randomization procedure performed by a blinded research assistant. At the end of the four weeks, 30 participants remained in the GCBT group and 28 in the WLC group. Measurement of heart rate variability and clinical symptoms were obtained at baseline and four weeks for both groups. After the quality control of HRV and considering the actual wearing situation of wearable devices, a total of 16 participants from the GCBT group and 17 participants from the WLC group were ultimately included in the study analysis.

This study was conducted in compliance with ethical standards and was approved by the Ethics Committee of Xinxiang Medical University (XYLL-2,020,235). All participants participated in this study voluntarily and signed the online informed consent before participation. All participants who completed the assessments were provided with their individual psychometric results.

### GCBT procedures

Treatment in the form of GCBT occurred over four sessions, each lasting 90 min, conducted across four consecutive weeks. The format included two face-to-face sessions and two online sessions. All GCBT modules were designed based on the cognitive behavioral model by Beck et al. [27], following the GCBT programmer (WY) registered psychologists with more than 3 years of experience, manualized intervention [28]. The intervention encompassed the following components: (a) Member introductions and understanding of depression, anxiety, and CBT. (b) Identification and exploration of distorted cognition and negative behaviors. (c) Acquisition of corrective skills to address distorted cognition. (d) Consolidation of cognitive changes and application of corrective skills. To support the research process, four local University College Student Volunteers (UCSVs) were recruited as research assistants and underwent a 1-day training session on data collection and participant management during the GCBT intervention period. After randomization, the GCBT group, were assigned to groups of six to ten participants, began the intervention immediately. The WLC group underwent a four-week waiting period and proceeded with treatment after the waiting period based on individual preference. Meanwhile, the WLC group had no scheduled contact with the project team, they can get help and support as needed. Throughout the entire intervention or waiting period, a psychological service hotline had been provided to prevent the occurrence of severe adverse events. Table 1 describes an overview of the treatment content.

**Table 1:** Description of GCBT treatment

Module	Theme	Main subject
1	Introduction to CBT	Member introductions and understanding of depression, anxiety, and CBT
2	delves into automatic thinking	Identification and exploration of distorted cognition and negative behaviors
3	Acquisition of corrective skills	Identifying and challenging negative automatic thinking and intermediate beliefs
4	Consolidation of CBT and application	Understanding core beliefs and mastering techniques for correction

### Data collection

During the four-week study period from 07:00 am to 21:00 pm participants were informed about and instructed to wear wearable wristbands. The collected data was stored on the device and uploaded to research servers every night, with text reminders sent to those who failed to upload on time. We utilized Huawei Band 6 devices with photoplethysmography (PPG) sensors (100 Hz) to record the physiological signals and calculate the continuous inter-beat interval and heart rates. Huawei smart wearable devices had previously been employed in previous clinical studies for cardiovascular disease [29–31]. We then used inter-beat interval records to calculate Heart rate variability (HRV) parameters, such as Standard Deviation of Normal-to-Normal Intervals (SDNN), Root Mean Square of Successive Differences (RMSSD) and so on. The devices also featured a 3-axis accelerometer to measure acceleration, which was used to calculate activity level as an indicator of overall movement, with daily step count serving as a proxy for physical activity.

### Data quality control

**Fig. 1:**
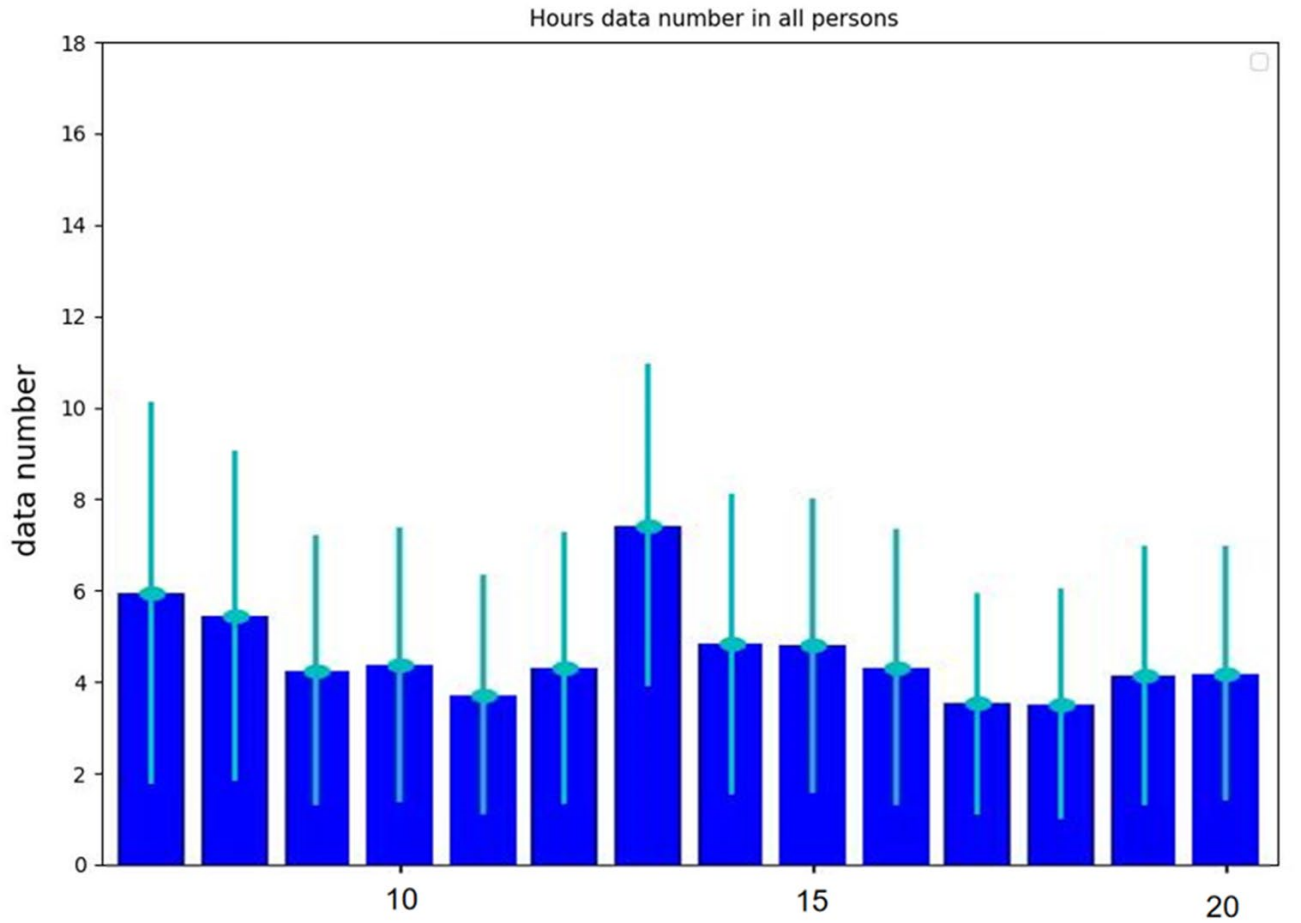
The hourly effective 5-minute sample numbers of the participants

To standardize the study, it is recommended to perform short-term measurement analysis of heart rate variability (HRV) using a continuous 5-minute sequence of R-R intervals (RRI, represent R-wave peak to R-wave peak intervals in electrocardiogram (ECG) records) to assess the temporal changes between successive heartbeats [32]. Previous studies indicate that physiological information obtained from the R-R intervals in electrocardiogram can similarly be derived from the pulse period of photoplethysmography (PPG), with a confirmed strong correlation between PPG-derived peak-to-peak intervals and ECG-derived R-R intervals [33, 34]. In this study, PPG-derived peak-to-peak intervals were used to represent R-R intervals. All returned data were analyzed, and the number of valid 5-minute samples per hour for each record was calculated separately, as shown in Fig. 1. Taking into account HRV was influenced by circadian rhythms, we made the final decision to focus on HRV measurements for all participants taken between 01:00 pm and 02:00 pm [35]. This time slot was chosen due to its highest data integrity, ensuring minimal disturbance and thus providing the most accurate and reliable data. In accordance with the accepted guidelines, we only included series with a minimum of 85.0% sinus beats in order to ensure data quality and reliability for our study [36–38]. In the heart rate variability records collected by our participants, only 16 participants from the GCBT group and 17 participants from the WLC group met the criteria and were retained for subsequent analysis.

### Data preprocessing

Prior to analysis, we utilized the HRV analysis toolkit (https://aurahealthcare.github.io/hrvanalysis/) to process R-R intervals, which underwent meticulous manual correction for ectopic beats and missing heartbeats [39]. In accordance with our criteria, whenever ectopic beat or missing heartbeats were detected, they were surgically removed and replaced with the mean of the two adjoining R-R intervals [21, 37]. HRV indices were computed in accordance with the recommendations outlined by the Task Force of the European Society of Cardiology and the North American Society of Pacing and Electrophysiology in 1996 [40]. These calculations encompassed time domain features, frequency domain features, and non-linear domain features of HRV for individuals.Time domain features included Mean HR, Min HR, Max HR, Median NN, SDNN, SDSD, NN20, NN50, PNN50, PNN20, RMSSD. Frequency domain features encompassed LF, HF, and the LH/HF ratio. Additionally, the non-linear domain feature known as the Triangular index was calculated. We calculated valid samples within this time period for one week and took their average as the heart rate variability (HRV) for the recent week. The data from the first week is considered as the baseline HRV. Similarly, HRV for the fourth week is calculated in the same manner (See Abbreviation Supplemental Table 1).

### Statistical analysis

In this study, we analyzed their data using IBM SPSS Statistics 25.0 software. To compare differences between groups at baseline, we employed various statistical tests, including Chi-square or Fisher’s tests for qualitative variables and Student’s t-test or Mann-Whitney test for quantitative variables. With “time” as the within-group factor and “group” as the between-group factor, we used repeated-measures analyses of variance (RMANOVA) to assess the effects of the intervention on outcome measures over time. The association between HRV, constructed a multiple linear Symptom and GCBT was analyzed in two stages. First, we employed Spearman rank correlations to explore the associations between clinical and functional indicators and HRV, with a significance threshold set at *p* < 0.05 to help explicate our results. Additionally, we performed regression model using relevant variables to investigate whether the baseline level of HRV can predict changes in depression and anxiety.

## Results

### Demographic characteristics of participants

**Fig. 2:**
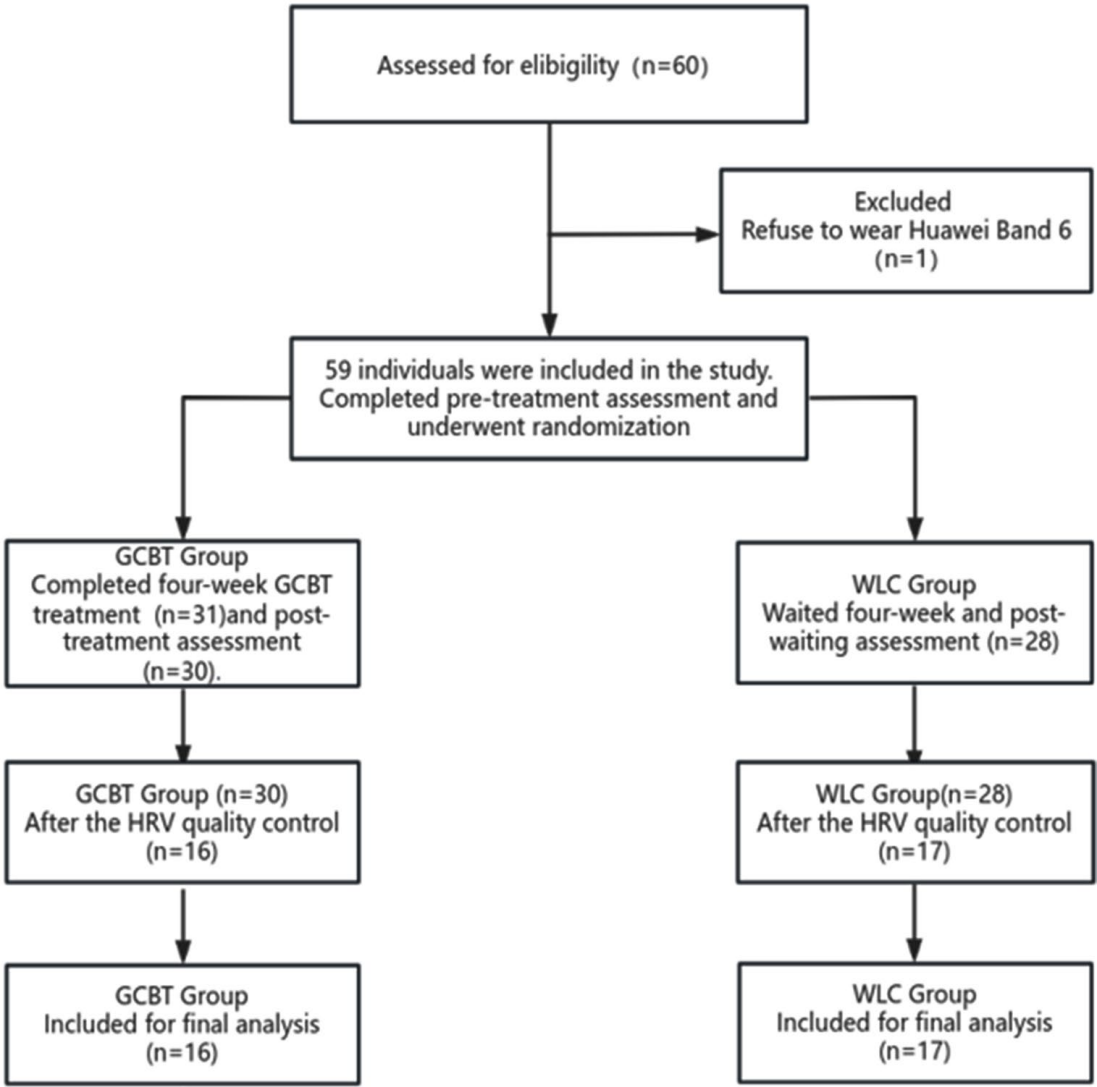
Participant flow and reasons for dropping out throughout the trial

After physiological signal data preprocessing and quality control, the analysis included 16 participants from the GCBT group and 17 participants from the WLC group. Please see Fig. 2 for details regarding participant flow and drop-out. There were no significant differences (*P* > 0.05) between groups in terms of sociodemographic in Table 2. In the other hand, there were no significant differences (*P* > 0.05) between groups in terms of heart rate variability in Table 3.

**Table 2:** The demographic and clinical characteristics of GCBT and WLC groups

	GCBT(*n* = 16)	WLC(*n* = 17)	T / χ2	p
**Mean age, years (SD)**	20.24(1.238)	20.82(3.644)	0.597	0.56
**Sex, n (%)**			0.4	0.55
Male	5(31.25)	6(35.29)		
Female	11(68.75)	11(64.71)		
**Smoking rules, n (%)**			2.3	0.13
Never smoke	14(87.50)	17(100.00)		
Sometimes smoke (cumulative smoking < 10 packs)	2(12.50)	0		
**Alcohol history, n (%)**			0.8	0.36
Never drink	11(68.75)	17(100.00)		
Drink occasionally (less than one time per week)	5(31.25)	0		
**BMI (kg/m** ^**2**^ **)**	20.25(1.238)	20.82(3.644)	0.597	0.56

**Table 3:** Comparison of outcome of HRV before and after the intervention and between two groups

Category	Variable	Pre/post	GCBT (*n* = 16)	WLC (*n* = 17)	P-value comparing before and after the intervention
Time domain	Mean HR	Before	72.51(5.09)	72.52(9.32)	0.998
		After	74.21(6.83)	72.22(8.81)	0.475
	Max HR	Before	93.29(5.74)	93.38(10.87)	0.979
		After	95.00(6.46)	92.21(11.19)	0.872
	Min HR	Before	47.72(2.57)	47.94(4.57)	0.868
		After	48.64(4.28)	27.96(4.50)	0.66
	Median NN	Before	836.70(64.39)	848.34(134.34)	0.756
		After	819.56(86.38)	849.94(109.57)	0.401
	SDNN	Before	88.24(19.23)	84.86(16.23)	0.588
		After	81.29(15.59)	83.56(22.51)	0.741
	SDSD	Before	55.64(14.17)	53.60(10.74)	0.643
		After	51.80(9.46)	53.13(13.07)	0.741
	NN 20	Before	120.51(37.51)	131.29(36.57)	0.41
		After	127.75(35.25)	132.56(47.91)	0.746
	PNN20	Before	35.67(11.75)	39.42(12.22)	0.377
		After	37.40(12.31)	39.40(15.20)	0.683
	NN 50	Before	43.54(22.73)	59.24(24.74)	0.494
		After	44.00(19.60)	50.96(34.11)	0.481
	PNN50	Before	12.97(6.89)	15.04(8.58)	0.452
		After	12.96(6.37)	15.12(10.11)	0.465
	RMSSD	Before	55.64(14.17)	53.60(10.74)	0.643
		After	51.80(9.46)	53.13(13.07)	0.741
Frequencial	LF	Before	81.43(4.15)	79.87(6.14)	0.402
		After	79.86(3.79)	79.62(6.90)	0.901
	HF	Before	18.57(4.15)	20.13(6.12)	0.402
		After	20.14(3.79)	20.38(6.90)	0.9
	LF/HF	Before	5.64(1.93)	5.50(2.50)	0.86
		After	5.01(1.45)	5.35(2.29)	0.617
Non linear	Triangular index	Before	11.86(2.61)	12.01(2.78)	0.87
		After	11.59(2.53)	12.13(3.28)	0.6

### GCBT intervention effect of clinical symptoms

Kolmogorov-Smirnov tests showed that HRV and scale scores were normally distributed. For these variables, we used two-tailed T-tests for analysis and found no group differences in HRV and symptom severity (PHQ, GAD, PSS) before intervention between the GCBT and WLC groups (*P* > 0.05). RMANOVA with intervention (GCBT or WLC group) as a within-subject factor was used to compare the treatment and wait-list control groups. Depression scores: The main effect of GCBT intervention was not significant, F = 0.335, *P* = 0.57, partial η2 = 0.11; the main effect of time was not significant, F = 3.51, *P* = 0.07, partial η2 = 0.11; the interaction effect of GCBT intervention and time was significant, F = 5.87, *P* = 0.02, partial η2 = 0.16. Anxiety scores: The main effect of GCBT intervention was not significant, F = 0.78, *P* = 0.38, partial η2 = 0.06; the main effect of time was not significant, F = 4.119, *P* = 0.05, partial η2 = 0.12; the interaction effect of GCBT intervention and time was significant, F = 4.69, *P* = 0.04, partial η2 = 0.135. Stress scores: The main effect of GCBT intervention was not significant, F = 0.009, *P* = 0.924, partial η2 = 0.001; the main effect of time was not significant, F = 0.396, *P* = 0.534, partial η2 = 0.013; the interaction effect of GCBT intervention and time was significant, F = 6.034, *P* = 0.020, partial η2 = 0.163 (Table 4).

**Table 4:** Comparison of outcome variables before and after the intervention and between two groups

Variable	GCBT group (*n* = 16)		WLC group (*n* = 17)		F			P	η2
	Pre-test	Post-test	Pre-test	Post-test	Group	Time	Group×time		
PHQ	5.75 ± 3.088	3.75 ± 2.324	4.12 ± 2.233	4.47 ± 2.324	0.335	3.51	5.24	0.03	0.15
GAD	4.06 ± 2.435	2.63 ± 1.784	2.53 ± 1.90	2.76 ± 2.386	0.78	4.119	4.69	0.038	0.135
PSS	20.06 ± 6.81	17.63 ± 1.784	16.59 ± 7.49	20.71 ± 7.597	0.009	0.396	6.034	0.020	0.163

Abbreviations: GCBT: Group cognitive behavioral therapy; WLC, wait-list control

PHQ-9: Patient Health Questionnaire 9-item; GAD-7: Generalized Anxiety Disorder Scale 7-item; PSS-14: Perceived Stress Scale 14-item

### Associations between clinical symptoms and heart rate variability

The correlation analysis revealed significant negative associations between HRV, heart rate (HR), and the scale scores of the PSS and PHQ. Specifically, the mean HR exhibited a negative correlation with both PHQ scores (rho=−0.313, *P* = 0.01) and PSS scores (rho=−0.313, *P* = 0.01). Similarly, the minimum HR showed negative correlations with PHQ scores (rho=−0.228, *P* = 0.048) and PSS scores (rho=−0.253, *P* = 0.03). Additionally, HFnu (high frequency normalized units) exhibited negative correlations with both PHQ scores (rho=−0.49, *P* < 0.001) and PSS scores (rho=−0.283, *P* = 0.02), while the correlation between LFnu and PHQ and PSS scores was found to be exactly opposite to that of HFnu. Lastly, the LF/HF ratio (representing the low frequency and high frequency ratio) demonstrated positive correlations with PHQ scores (rho = 0.49, *P* < 0.001) and PSS scores (rho = 0.30, *P* = 0.01). No statistically significant correlation was found between HRV, HR, and GAD scores (Table 5).

**Table 5:** Correlation analysis results of HRV, PSS and PHQ-9 scores

Symptom measures	Heart rate variability									
	Mean hr		Min hr		LF/HF		LFnu		HFnu	
	rho	P	Rho	P	rho	P	rho	P	rho	P
PHQ-9 score	−0.313	0.01	−0.228	0.048	0.49	< 0.001	0.49	< 0.001	−0.49	< 0.001
PSS-14 score	−0.313	0.01	−0.253	0.02	0.30	0.01	0.283	0.02	−0.283	0.02

PHQ-9: Patient Health Questionnaire 9-item; PSS-14: Perceived Stress Scale 14-item

### Associations between treatment effect and baseline heart rate variability

**Fig. 3:**
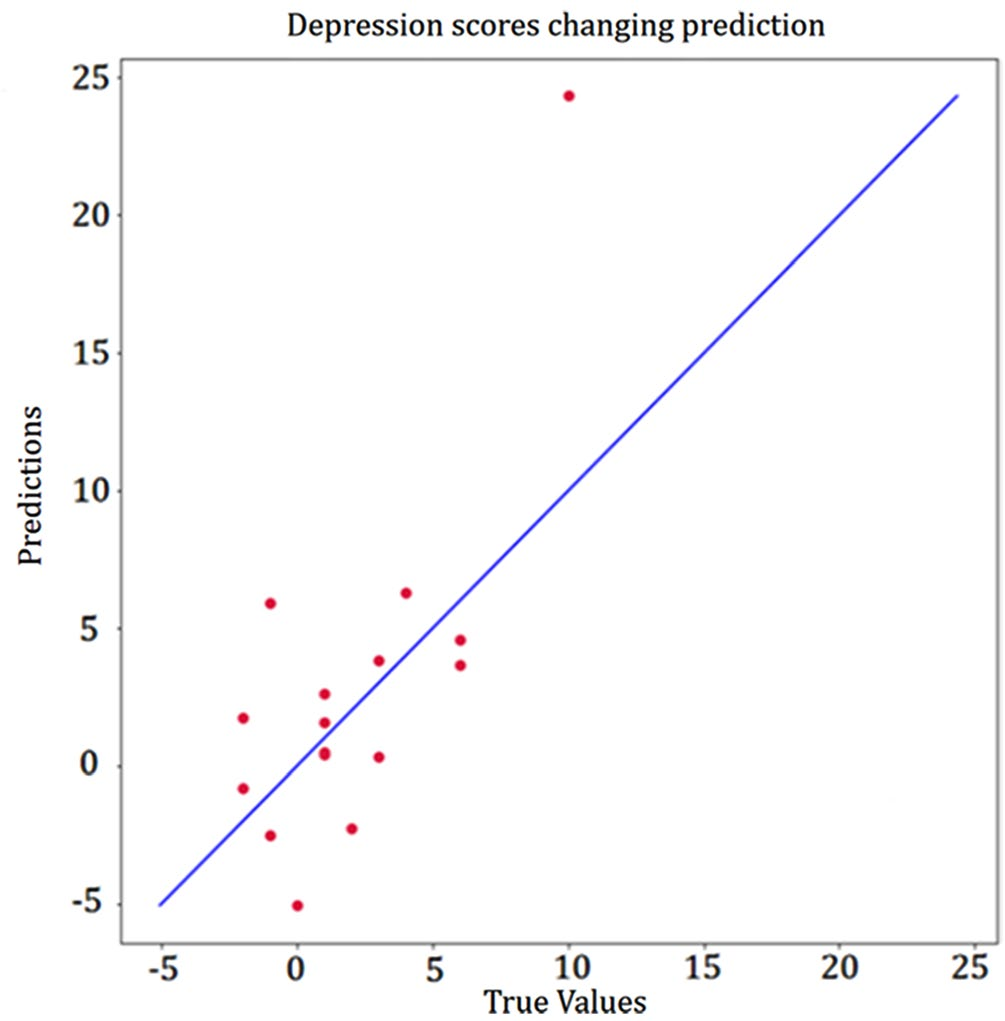
Correlation of HRV with change in depression symptoms (PHQ-9). ^a^The red dots represent each participant. The X-axis indicates the actual improvement in depressive symptoms. The Y-axis represents the predicted improvement in depressive symptoms

**Fig. 4:**
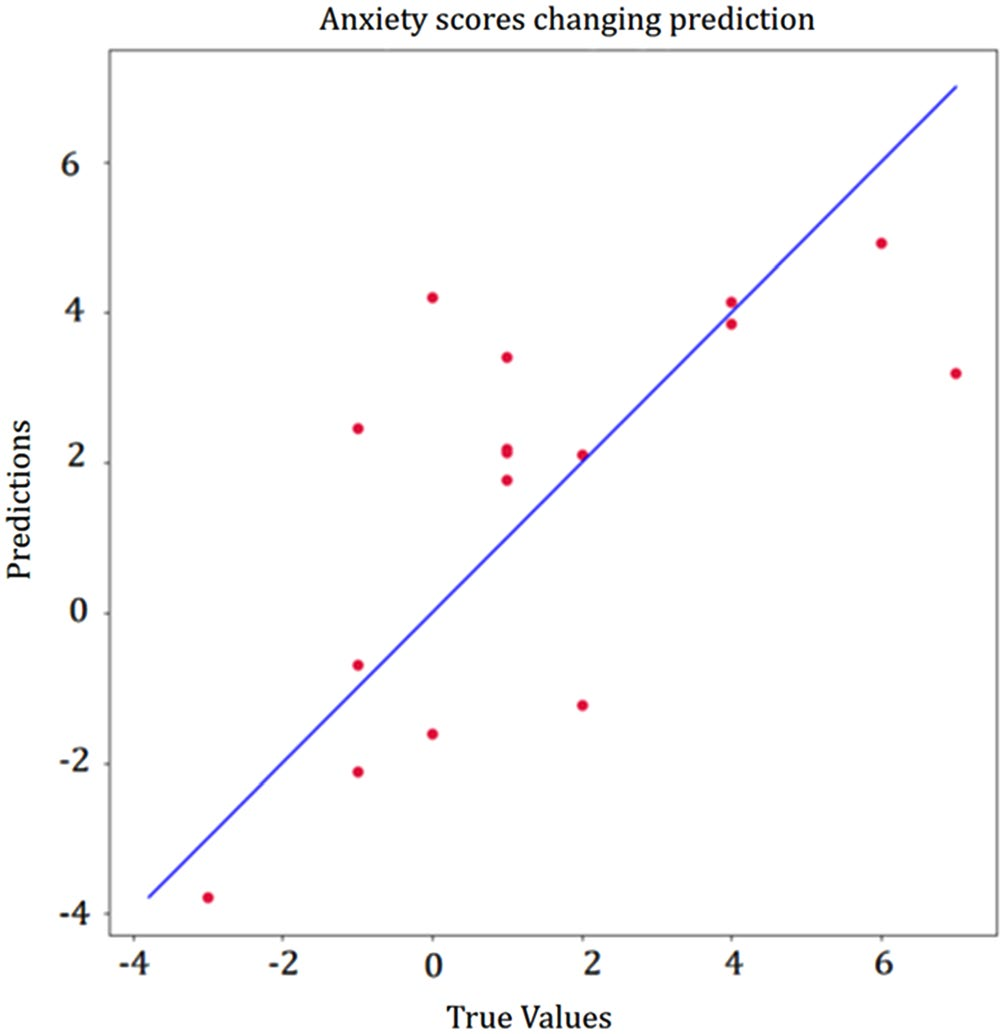
Correlation of HRV with change in anxiety symptoms (GAD-7). ^a^The red dots represent each participant. The X-axis indicates the actual improvement in depressive symptoms. The Y-axis represents the predicted improvement in depressive symptoms

From pre- to post-treatment, participants’ improvement in depressive symptoms was positively correlated with baseline HRV: NN20 (rho = 0.55, *P* = 0.03), Median NN (rho = 0.499, *P* = 0.049), PNN20 (rho = 0.547, *P* = 0.03), while Mean HR (rho=−0.507, *P* = 0.045), Min HR (rho=−0.551, *P* = 0.03) the negative correlation with declination of depression scores. The absolute difference in anxiety scores was also significant correlated with baseline HRV: SDNN (rho = 0.548, *P* = 0.03), SDSD (rho = 0.532, *P* = 0.03), NN50 (rho = 0.593, *P* = 0.02), PNN50 (rho = 0.612, *P* = 0.01), PNN20 (rho = 0.618, *P* = 0.01), RMMSD (rho = 0.532, *P* = 0.03), Min HR (rho=−0.734, *P* = 0.001), Triangular index (rho = 0.682, *P* = 0.004). A multiple linear regression model was used to explore the relationship between HRV and the anti-depressive and anti-anxiety efficacy of GCBT treatment. We screened for HRV features related to GCBT efficacy and constructed a multiple linear regression model using backward selection to ultimately establish a final model containing independent variables of HRV features, gender, age and BMI, and activity level (showed in Supplementary Table 2). The results showed that HRV was a significant predictor of GCBT anti-depressive and anti-anxiety efficacy after controlling for the effects of other independent variables. We found the multiple HRV features significantly predicted depressive symptoms changes (F = 7.308, *P* = 0.02, R2 = 0.936) (Fig. 3). We found that predicted changes in anxious symptoms were also significant (F = 13.849, *P* = 0.002, R2 = 0.954) (Fig. 4). Detailed information about HRV parameter in the models can be found in Supplementary Table 3.

## Discussion

In this preliminary study, we assess the efficacy of GCBT as a treatment for depression and anxiety. Our analysis revealed significant and clinically meaningful reductions in depression symptoms (PHQ scores) and anxiety symptoms (GAD scores) relative to baseline, post-intervention at 4 weeks. Additionally, we assessed the association between autonomic abnormalities and symptoms of depression, anxiety, and pressure level. Our findings suggest that the reduction in depression and anxiety scores positively correlated with baseline HRV levels. To our knowledge, this is the first study to describe the efficacy of depression and anxiety treatment using passively collected data from wearable devices, providing a basis for future research on potential predictors of anti-depression and anti-anxiety treatment response.

Although a study by Balogh et al. (1993) had previously found no correlation between pre-treatment resting-state HRV and improvement in depressive symptoms [41], we speculate that this conclusion may have stemmed from inadequate sample size and dependence on laboratory data. To mitigate these limitations, participants in our study were required to wear wristbands for four weeks to collect personalized data. By collecting data passively from wearable devices over the course of a week, we avoided the systematic errors and laboratory effects present in previous studies that recruited patients and collected 5-minute HRV in the laboratory. This approach is more stable and reflects a resting HRV index that is closer to real-world conditions. In contrast, most previous studies used electrocardiograms to measure HRV. ECG electrodes are typically attached to the body with adhesive, which can cause skin damage related to medical adhesive, especially if worn for extended periods [42].

In our study, participants with lower HRV reported higher depression scores. Most diagnosed mental illnesses often exhibit lower HRV [43]. Although participants in our experiment were in state of subthreshold mental health with depression and anxiety, their HRV followed this pattern, indicating that even in the early stages of MDD or ANX, HRV has already begun to affect their onset and development. Abnormal functioning of the autonomic nervous system may develop into psychopathology. HRV can serve as a biological marker for predicting depression states.

Our study revealed a consistent positive correlation between HRV indices and self-reported depression and stress scores in patients. HRV serves as an indicator of an individual’s ability to adapt physiologically to changing internal and environmental demands, reflecting their overall quality factor [44]. Lower HRV is associated with increased vulnerability to stress and reduced coping abilities. It also indicates heightened control by the sympathetic nervous system, which can lead to difficulties in emotional regulation and increased impulsivity.

Additionally, higher resting HRV has been linked to various psychological processes, including more flexible and adaptive emotion regulation and recovery in response to threat [45–47]. Notably, pre-treatment HRV predicted outcomes of GCBT, with higher HRV predicting better treatment outcomes and lower HRV predicting poorer outcomes. Given the current results that individuals with higher HRV indexes a more flexible or responsive physiological system that may benefit more strongly from GCBT treatment. Interestingly, we found that HRV time-domain and non-linear indicators are more robust and closely related to treatment effects, while frequency-domain indicators and HR appear to reflect the participant’s state at the time of measurement, including their mood or stress levels.

After four weeks of cognitive-behavioral therapy, depressive and anxious score improved in GCBT group, as did the HF/LF ratio, we believe that cognitive-behavioral therapy may partially achieve its efficacy through improving HRV. This is based on the fact that the neural mechanism of CBT treatment mainly targets abnormal neural circuits in depression by regulating core targets such as the amygdala and anterior cingulate cortex [48]. The therapeutic effect of CBT is related to task-related activation, especially activation of the amygdala and subgenual ACC (sgACC), while sgACC is closely related to depression and autonomic nervous system activity. Increased connectivity patterns between affective (sgACC and amygdala) and cognitive control (dorsolateral prefrontal cortex and Temporal parietal junction) networks before and after CBT are associated with successful CBT treatment [49]. Connectivity in these regions can predict CBT treatment response in depressed adolescents. This indicates that successful treatment may be related to the reconfiguration of previously disordered connectivity patterns and provides neuroimaging evidence that HRV can be used as a biological marker to predict the efficacy of GCBT.

This study has several limitations that should be considered when interpreting the results. One limitation of this study is the small sample size. Although we have addressed this to some extent by collecting passive data over an extended period, there remains a possibility of chance occurrences. This could potentially impact the reliability and generalizability of the study findings. In future research, we aim to increase the sample size to further validate our findings. Additionally, a subset of participants was excluded from the data analysis, prompting us to delve into the intricacies of this phenomenon. Possible explanations include: WLC participants with higher depression and anxiety scores may have dropped out during the waiting period due to non-compliance with wristband requirements. Participants in the GCBT group, who had relatively milder clinical symptoms, may have exhibited poorer compliance with wearable devices and treatment. Finally, we controlled for activity level confounding factors in subsequent analyses even though we cannot guarantee that participants were in an absolute resting state. We believe that our definition of resting state may represent most states in daily life for college students. Meanwhile, the potential bias conducted by non-continuous data is mitigated due to our coverage requirement.

## Conclusion

This exploratory pilot data tentatively supports the hypothesis that treatment outcomes of GCBT for depressive and anxious symptoms are associated with autonomic nervous system function. Smart wearable devices have emerged as low-cost, non-invasive, and convenient tools for monitoring and predicting mental health. Based on data collected from smart devices, HRV can be an easily measurable and objective predictor of treatment response to GCBT for individuals with anxiety and depression. It is therefore worth considering in the context of the high cost of treatment and the longer time it takes to transmit more sophisticated brain-based and other biological assessments. Future research could consider increasing sample size or exploring different treatment methods, such as Transcranial Direct Current Stimulation (tDCS) or repetitive Transcranial Magnetic Stimulation (rTMS), to assess whether low HRV can serve as an indicator of patient heterogeneity, thereby promoting personalized intervention treatments.

### Electronic supplementary material

Below is the link to the electronic supplementary material.

Supplementary Material 1

## Data Availability

The authors do not have the right to share any data information as per the ethics committee rules and regulations but are available upon a reasonable request from the corresponding author (FW).
