# Supplementary material for: Prediction of the efficacy of group cognitive behavioral therapy using heart rate variability based smart wearable devices: a randomized controlled study
Source: BMC Psychiatry. 2024 Mar 6;24:187. doi: 10.1186/s12888-024-05638-x (PMC10916138; doi:10.1186/s12888-024-05638-x)
Supplement: Supplementary file 1 — Supplementary Material 1 [file 12888_2024_5638_MOESM1_ESM.pdf]

Supplemental Table 1:HRV and definitions

HRV	Definitions
SDNN	Standard Deviation of NN Intervals
SDSD	Standard Deviation of Successive Differences
NN50	Number of Interval Differences of NN Intervals greater than 50 ms
PNN50	Percentage of NN50 divided by the total number of NN intervals
NN20	Number of Interval Differences of NN Intervals greater than 20 ms
PNN20	Percentage of NN20 divided by the total number of NN intervals
RMSSD	Root Mean Square of Successive Differences
Median NN	Median of NN Intervals
Mean HR	Mean Heart Rate
Max HR	Maximum Heart Rate
Min HR	Minimum Heart Rate
LF	Low Frequency - Represents the power in the low-frequency range (usually 0.04 to 0.15 Hz) of the heart rate variability spectrum
HF	High Frequency - Represents the power in the high-frequency range (usually 0.15 to 0.4 Hz) of the heart rate variability spectrum
LH/HF ratio	Low Frequency to High Frequency ratio - Calculated by dividing the power in the low-frequency range by the power in the high-frequency range
LFnu	the normalized contribution of low-frequency (LF) power to the total power of the heart rate variability spectrum
HFnu	the normalized contribution of high frequency (HF) power to the total power of the heart rate variability spectrum
Total Power	Represents the total power of the heart rate variability spectrum across all frequency ranges
Triangular Index	a metric used to evaluate the evenness or equitability of the distribution of HRV values

Supplemental Table 2 Correlation analysis of clinical symptom improvement and heart rate variability

HRV Variable	PHQ-9 changes		GAD-7 changes	
	rho	P	rho	P
SDNN	0.159	0.556	0.548	0.028
SDSD	0.172	0.523	0.532	0.034
NN50	0.275	0.303	0.593	0.016
PNN50	0.317	0.232	0.612	0.012
NN20	0.55	0.027	0.553	0.026
PNN20	0.547	0.028	0.618	0.011
RMSSD	0.172	0.523	0.532	0.034
Median NN	0.499	0.049	0.401	0.124
Mean HR	-0.507	0.045	-0.408	0.117
Min HR	-0.551	0.027	-0.734	0.001

Triangular Index	0.462	0.072	0.682	0.004

Supplemental Table 3: Detailed parameter information of models predicting Depression and Anxiety improvement using heart rate variability.

HRV Variable	Depression model			Anxiety model		
	**β**	**P**	**P_{FDR}**	**β**	**P**	**P_{FDR}**
SDNN	-0.269	<0.001	0.001	-0.281	<0.001	<0.001
Min HR	-3.264	<0.001	<0.001	-4.215	<0.001	0.001
NN 50	2.355	0.001	0.001	0.394	0.174	0.218
PNN50	-7.678	0.001	0.001	-1.208	0.203	0.203
Median NN	NA	NA	NA	-0.102	<0.001	0.001
